# Commentary: A comprehensive review of diagnostic approaches for hepatitis D

**DOI:** 10.3389/fmolb.2026.1855687

**Published:** 2026-06-12

**Authors:** Sally A. Baylis, Gabriele Unger, Karin J. Metzner

**Affiliations:** 1 Viral Safety Section, Division of Infectious Diseases, Paul-Ehrlich-Institut, Langen, Germany; 2 WHO Collaborating Center for Quality Assurance of Blood Products and In Vitro Diagnostic Devices, Paul-Ehrlich-Institut, Langen, Germany; 3 EU Reference Laboratory for In Vitro Diagnostic Medical Devices, Paul-Ehrlich-Institut, Langen, Germany

**Keywords:** HDV, hepatitis delta virus, international standard, NAAT, NAT, WHO, World Health Organization

## Introduction

We read with interest the review by Liu et al. (2025) on diagnostic testing for hepatitis delta virus (HDV). Given the burden of disease, the authors highlight the urgent need to establish standardized protocols and international standards for the detection of HDV to ensure appropriate management and timely treatment of hepatitis delta infections. The World Health Organization (WHO) has recently prioritized the inclusion of HDV in the WHO Model List of Essential *In Vitro* Diagnostics, including serology, rapid tests, and nucleic acid amplification technique (NAT or NAAT) assays (World Health Organization, 2024). Liu et al. (2025) refer to the 1st WHO International Standard (IS) for HDV RNA for NAT-based assays (code number 7657/12); however, the authors incorrectly state that the 1st WHO IS is no longer available.

## Availability and characteristics of the 1st World Health Organization international standard for hepatitis delta virus RNA

The 1st WHO IS was prepared using viremic plasma from a Turkish patient infected with genotype 1 HDV. Similar to other WHO ISs for NAT, the choice of strain used to prepare the standard was based on global prevalence (Baylis et al., 2019). The 1st WHO IS was established by the WHO Expert Committee on Biological Standardization (ECBS) in October 2013 (Chudy et al., 2013). The international study to evaluate the IS demonstrated that reporting of viral loads varied by up to 1,000-fold; however, when results were expressed against the WHO IS, there was a marked improvement in the agreement between laboratories. It is important to clarify that this standard remains available and is currently distributed by the Paul-Ehrlich-Institut (PEI) in Langen, Germany. As a WHO Collaborating Center for Quality Assurance of Blood Products and *in vitro* Diagnostic Devices (IVDs), the PEI was responsible for the development of this standard and continues to act as the custodian laboratory, managing its storage and distribution worldwide. Characteristics of the WHO IS are listed in Table 1. Although the sequence of the 1st WHO IS was misreported in the original study (Chudy et al., 2013), this issue was later identified during analysis by Pyne et al. (2017). The accession number HQ005369 correctly identifies the HDV strain used to prepare the standard (Baylis et al., 2019; Pyne et al., 2017).

**TABLE 1:** Characteristics of the 1st World Health Organization international standard for hepatitis delta virus RNA for nucleic acid amplification technique assays.

Name	1st World Health Organization international standard for hepatitis delta RNA for NAT-based assay
Product code number	7657/12
Genotype	1
Accession number	HQ005369
Concentration	575,000 international units per ml*
Contents	Freeze-dried residue of 0.5 mL of plasma
Availability	www.pei.de

NAT, nucleic acid amplification technique.

*Concentration after reconstitution in 0.5 mL of molecular grade water.

## Discussion

Since 2013, the WHO IS has been widely used and is intended primarily for the calibration of secondary standards in International Units (IU). Diagnostic assays for HDV are included in the European Union (EU) *In Vitro* Diagnostic Regulation (IVDR 2017/746). As high-risk Class D devices, these tests require mandatory performance verification and batch testing by EU Reference Laboratories, including the PEI, prior to market release. Under EU Regulation 2022/1107, specific quality standards or “common specifications” are mandatory for devices that detect or measure HDV markers, including viral RNA (European Commission, 2022). These regulations define essential assay performance criteria, including diagnostic sensitivity/specificity and genotype coverage. Analytical sensitivities and linear ranges of HDV NAT assays are determined by dilution of the 1st WHO IS (or a traceable equivalent). Furthermore, external quality assessment providers increasingly request the reporting of HDV RNA loads in IU as standard (Baylis et al., 2019).

Studies have demonstrated that the 1st WHO IS has helped improve comparability of HDV RNA measurement across various laboratories and methods and is commutable with clinical samples (Chudy et al., 2013; Stelzl et al., 2021). In 2023, the European Association for the Study of the Liver (EASL) published the 1st Clinical Practice Guidelines for HDV (European Association for the Study of the Liver, 2023). It is important to note that the EASL guidelines state the following: “*In both clinical trials and practice, HDV RNA should be quantified by a reference laboratory using well-standardized, validated assays, and the results should be given in IU/mL to improve precision and comparability across laboratory test systems.”* Given the EASL recommendation to report HDV RNA loads in IU/mL, it is essential that laboratories continue to use the 1st WHO IS for HDV RNA to ensure harmonization of results and best clinical practice moving forward. These efforts are underpinned by regulations in Europe and the anticipated expansion of the WHO Model List of Essential *In Vitro* Diagnostics.

Details for ordering the 1st WHO IS for HDV RNA are available on the PEI website (www.pei.de).
